# *TYMS* 3′-UTR Polymorphism: A Novel Association with FOLFIRINOX-Induced Neurotoxicity in Pancreatic Cancer Patients

**DOI:** 10.3390/pharmaceutics14010077

**Published:** 2021-12-29

**Authors:** Marina Emelyanova, Ilya Pokataev, Igor Shashkov, Elena Kopantseva, Vladimir Lyadov, Rustam Heydarov, Vladimir Mikhailovich

**Affiliations:** 1 Engelhardt Institute of Molecular Biology, Russian Academy of Sciences, 119991 Moscow, Russia; emel_marina@mail.ru (M.E.); igorshashkov@bk.ru (I.S.); y.kopantseva@gmail.com (E.K.); rustam.heydarov@gmail.com (R.H.); 2 Department of Oncology, Moscow Clinical Oncology Hospital No.1, Moscow City Health Department, 105005 Moscow, Russia; PokataevIA@zdrav.mos.ru (I.P.); vlyadov@gmail.com (V.L.); 3 Federal Research Centre ‘Fundamentals of Biotechnology’, Russian Academy of Sciences, 119071 Moscow, Russia; 4 Department of Oncology and Palliative Medicine, Russian Medical Academy of Continuous Professional Education, 123242 Moscow, Russia; 5 Department of Oncology, Novokuznetsk State Institute for Continuous Medical Education, 654005 Novokuznetsk, Russia

**Keywords:** *TYMS*, *GSTP1*, rs11280056, pancreatic ductal adenocarcinoma, drug-induced toxicity, FOLFIRINOX, genotyping, microarray

## Abstract

Pancreatic ductal adenocarcinoma (PDAC) is a highly fatal malignancy that has the worst 5-year survival rate of all of the common malignant tumors. Surgery, chemotherapy, and/or chemoradiation remain the main tactics for PDAC treatment. The efficacy of chemotherapy is often compromised because of the substantial risk of severe toxicities. In our study, we focused on identification of polymorphisms in the genes involved in drug metabolism, DNA repair and replication that are associated with inter-individual differences in drug-induced toxicities. Using the microarray, we genotyped 12 polymorphisms in the *DPYD*, *XPC*, *GSTP1*, *MTHFR*, *ERCC1*, *UGT1A1*, and *TYMS* genes in 78 PDAC patients treated with FOLFIRINOX. It was found that the *TYMS* rs11280056 polymorphism (6 bp-deletion in *TYMS* 3′-UTR) predicted grade 1–2 neurotoxicity (*p* = 0.0072 and *p* = 0.0019, according to co-dominant (CDM) and recessive model (RM), respectively). It is the first report on the association between *TYMS* rs11280056 and peripheral neuropathy. We also found that PDAC patients carrying the *GSTP1* rs1695 GG genotype had a decreased risk for grade 3–4 hematological toxicity as compared to those with the AA or AG genotypes (*p* = 0.032 and *p* = 0.014, CDM and RM, respectively). Due to relatively high *p*-values, we consider that the impact of *GSTP1* rs1695 requires further investigation in a larger sample size.

## 1. Introduction

Pancreatic ductal adenocarcinoma (PDAC) is a highly fatal malignancy that has the worst 5-year survival rate of all of the common malignant tumors [1,2]. Most patients with PDAC are asymptomatic until the disease reaches an advanced, and often unresectable, stage. Less than one-third of patients are only candidates for surgical resection at the time of their diagnosis, and surgery, chemotherapy, and/or chemoradiation remain the main tactics for treatment [3]. Despite progress in surgical procedures, the tumor recurrence rate exceeds 60% after radical surgical resection, and patients are further managed by chemotherapy [4,5].

Adjuvant chemotherapy may significantly improve disease-free survival (DFS), progression-free survival (PFS), and overall survival (OS). The current standard duration of adjuvant chemotherapy for PDAC is six months. A combination therapy of FOLFIRINOX (5-fluorouracil, leucovorin, irinotecan, and oxaliplatin) has become one of the most common regimen for both (neo)adjuvant and advanced pancreatic cancer treatment [6,7]. In the neoadjuvant setting, the FOLFIRINOX regimen administered with or without radiotherapy was associated with improved tumor down-staging, converting initially unresectable tumors into successful resections with R0 margins in borderline resectable and locally advanced PDAC patients [8,9].

The efficacy of chemotherapy is often compromised because of the substantial risk for severe toxicities. Drug-induced adverse events (AEs) are responsible for treatment delay, reduction, cessation, or even the death of a patient. In particular, the FOLFIRINOX group has increased rates of grade 3–4 toxicities, especially neutropenia, diarrhea, and peripheral neuropathy [10].

Although the majority of cases of severe toxicity remain unexplained, a large subset of these AEs is associated with polymorphisms in metabolic, DNA replication and repair genes that are responsible for the differences in inter-individual drug response and toxicity. The FDA provides specific information regarding therapeutic management for some pharmacogenetic associations. However, most of the polymorphic variations have not been sufficiently evaluated in terms of the impact of genetic testing on therapeutic effectiveness or increased risk of specific AEs [11].

In the present work, we focused on the identification of polymorphisms in the genes involved in drug metabolism, DNA repair and replication that are associated with a substantial rate of toxicities. We genotyped 12 polymorphisms (*DPYD* rs3918290, rs75017182, rs55886062, rs67376798, rs2297595, *XPC* rs2228001, *GSTP1* rs1695, *MTHFR* rs1801133, *ERCC1* rs3212986, rs11615, *UGT1A1* rs3064744, and *TYMS* rs11280056) using microarray-based analysis in a cohort of PDAC patients treated with FOLFIRINOX.

It is crucial to be aware of genetic biomarkers that are predictive or prognostic with respect to drug-induced toxicity, since it may aid in treatment selection and allow clinicians to improve treatment outcome for every individual patient.

## 2. Materials and Methods

### 2.1. Patients

A total of 78 patients affected by pancreatic ductal adenocarcinoma and treated at the N. N. Blokhin Cancer Research Center (Moscow) with FOLFIRINOX between 2019 and 2020 were enrolled in the study. Main inclusion criteria were: PDAC histology, treatment with FOLFIRINOX regimen, availability of data regarding toxicity, and availability of blood samples.

All patients started their treatment with standard dosage. Dose reductions were made according to local practice. In general, any grade 3–4 adverse events and grade 1–2 neurotoxicity that caused a delay of the next cycle resulted in approximately 20% dose reduction for subsequent cycles. Patient characteristics are shown in Table 1.

The research was carried out in accordance with the Declaration of Helsinki and followed Good Clinical Practice guidelines. Approval of the study was obtained from the Ethics Committee of N. N. Blokhin Cancer Research Center in Moscow (Protocol code No 5, Date of approval: 27 May 2019). All subjects were required to read, understand, and sign an IRB-approved informed consent form. Laboratory investigators were blinded to the clinical information on patients’ data.

### 2.2. Assessment and Management of Chemotherapy Toxicity

A number of hematologic toxicities (anemia, leukopenia, neutropenia, and thrombocytopenia) and non-hematologic toxicities (asthenia, diarrhea, mucositis, nausea, stomatitis, vomiting, hepatic, skin, and neurotoxicity) were ascertained at the beginning of each chemotherapy cycle using Common Toxicity Criteria for Adverse Events (CTCAE), version 5.0 [12]. During the course of the treatment, all AEs were monitored, and maximum toxicity grade was reported.

### 2.3. Clinical Specimens and DNA Isolation

Between 3 and 5 mL of venous blood was collected in EDTA-containing tubes. The samples were stored at +4 °C during 3–10 days before DNA isolation. A QIAamp DNA Micro Kit (Qiagen, Hilden, Germany) was used to isolate DNA, according to the manufacturer’s instructions.

### 2.4. Control Samples

A total of 38 DNA samples with known genotypes were used as controls to verify the specificity of the microarray interrogating probes. The control sample panel included WT DNA samples as well as DNA samples harboring all corresponding variations to be analyzed. The control samples had been extracted from the whole blood earlier and had undergone preliminary genotype identification by Sanger sequencing.

### 2.5. Sequencing

Sequencing was performed on the Applied Biosystems 3730 DNA Analyzer (Applied Biosystems, Carlsbad, CA, USA), using the ABI PRISM^®^ BigDye™ Terminator v. 3.1 Kit (Applied Biosystems).

### 2.6. Oligonucleotide Probes, Primers, and Microarray Fabrication

Oligonucleotide probes for immobilization on a microarray support and PCR primers for multiplex amplification were synthesized using an automated 394 DNA/RNA synthesizer (Applied Biosystems). Oligonucleotides were designed with Oligo v. 6.31 (Molecular Biology Insights, Cascade, CO, USA) and BioEdit v. 7.09 software (Ibis Biosciences, Carlsbad, CA, USA). The oligonucleotide probes had a spacer with a free amino group 3′-Amino-Modifier C6 CPG 500 (Glen Research, Sterling, VA, USA) for subsequent copolymerization with microarray hydrogel components. The LNA residues (Qiagen, Hilden, Germany) were incorporated into *UGT1A1* genotyping probes to increase the specificity of hybridization analysis [13]. The nucleotide sequences of the probes and primers are shown in Tables S1 and S2, respectively. Biological microarrays were manufactured by the procedure described in detail earlier [14]. Each gel pad was in triplicate to improve the reliability of the analysis.

### 2.7. Genotyping of DNA Samples by Hybridization on Microarrays

DNA samples for hybridization on the microarray were prepared using two-stage asymmetric multiplex PCR. The reaction mixture contained 0.25 mM each of dATP, dCTP, dGTP, 0.125 mM of dUTP and dTTP (Evrogene, Moscow, Russia), 6.6 μM of Sulfo-Cyanine 5 dUTP (Lumiprobe, Moscow, Russia), 2.5 U *Taq* DNA polymerase (Qiagen, Hilden, Germany), 1 μL of primer mix containing 1 pmol/μL of each specific primer, 1 pmol/μL forward adapter primer and 100 pmol/μL reverse adapter primer, 3.3 μL 10 × PCR Buffer (Qiagen), 5.1 mM MgCl_2_ (Qiagen), and at least 10 ng of genomic DNA in a final volume of 33 μL. Amplification was performed on a C1000 Touch Thermal Cycler (BioRad, Hercules, CA, USA). The reaction mixture was incubated at 95 °C for 10 min followed by 25 cycles of 30 s at 95 °C, 70 °C, and 72 °C at the first PCR stage and then 49 cycles of 30 s at 95 °C, 54 °C, and 72 °C at the second PCR stage. Finally, the mixture was incubated for 5 min at 72 °C.

The mixture for hybridization analysis contained 33 μL of the PCR reaction mixture and 9 μL of a solution containing 4 M guanidine thiocyanate, 200 mM HEPES, and 20 mM EDTA (Sigma-Aldrich, St. Louis, MO, USA), pH 7.5. The resulting mixture was infused into a microarray chamber and incubated for 6 h at 37 °C. After hybridization, the microarray was washed twice with distilled water for 30 s at 37 °C and then dried.

### 2.8. Image Acquisition and Processing

Hybridization images were acquired and processed using a fluorescence analyzer setup with specialized software “ImaGel Studio” (Biochip-IMB, Moscow, Russia). To evaluate the discriminating ratio of hybridization probes, fluorescence signal intensities were acquired in each gel pad and processed using the software. The fluorescence signals produced by the microarray gel elements were used as the input data as follows: *J_m_ = (I_m_ − I*_0_*)/(B_m_ − I*_0_*)*, where *I_m_* was the fluorescence signal intensity per unit area in the internal part of a gel element, *B_m_* was the counterpart background intensity, *I*_0_ was the dark current in the charge-coupled device (CCD), and *m* was the gel element number. As every gel pad was in triplicate, the signal intensities were averaged.

Identification of single nucleotide variation (SNV) and deletions was based on differences in fluorescence signals acquired from gel pads bearing immobilized oligonucleotide probes to the major and minor allele variants. If the target contains any polymorphisms, a corresponding gel pad in which a perfect hybridization duplex has formed results in a significant increase in its fluorescence intensity.

### 2.9. Validation of the Microarray

The specificity of microarray genotyping was verified by comparative analysis with Sanger sequencing. The interrogating oligonucleotide probes corresponding to every allele variant were validated by hybridization of pre-sequenced samples. To be sure that signal intensity ratios are reproducible from one assay to another, each unique control genotype was analyzed three times.

### 2.10. Statistical Analysis

We classified the patients enrolled in the study into three genotype groups: individuals with the more frequent homozygous or a major genotype (AA), the heterozygous genotype (Aa), and the minor homozygous genotype (aa). The influence of each genotype on endpoints was estimated in accordance with three genetic models: (i) the co-dominant model, in which every effect of Aa and aa genotypes compared to AA were estimated; (ii) the dominant model, which assumes that the presence of one or two minor alleles has an equal effect, therefore, patients with Aa and aa variants were grouped together and compared to patients with the AA genotype; (iii) the recessive model, which supposes that the only one minor allele does not affect the clinical endpoints significantly. This model was used to estimate the effect of the aa genotype compared to Aa or AA genotypes pooled together. Testing for Hardy-Weinberg equilibrium was applied for detecting genotyping errors.

Two-sided Pearson’s χ2 tests and Cochran-Armitage trend tests were used to determine correlations between the qualitative characteristics. IBM SPSS Statistics software, version 26 (IBM Corp., Armonk, NY, USA) was used to perform statistical analysis.

## 3. Results

### 3.1. Design of Multiplex PCR

A single tube asymmetric multiplex PCR was developed for simultaneous amplification of 12 target sequences of *DPYD* rs3918290, rs75017182, rs55886062, rs67376798, rs2297595, *XPC* rs2228001, *GSTP1* rs1695, *MTHFR* rs1801133, *ERCC1* rs3212986, rs11615, *UGT1A1* rs3064744, and *TYMS* rs11280056. Primers containing specific and universal sequences complimentary to adapter primers were designed for target amplification on the first PCR stage (Table S1). The universal sequence part of forward and reverse primers was different. In the first PCR stage, targets of interest were amplified in a standard exponential manner, and the resulting PCR products contained universal sequences at their 5′ and 3′-ends. Shorter universal adapter primers were used in the second asymmetric PCR stage. At that stage, the annealing temperature was lower, and the concentration of universal reverse primers was significantly higher (see Materials and Methods). Due to this methodical approach, the reaction yielded predominantly single-stranded and fluorescently labeled amplification products suitable for subsequent hybridization analysis.

### 3.2. Microarray for Genotyping Analysis

A microarray for the simultaneous identification of *DPYD* rs3918290, rs75017182, rs55886062, rs67376798, rs2297595, *XPC* rs2228001, *GSTP1* rs1695, *MTHFR* rs1801133, *ERCC1* rs3212986, rs11615, *UGT1A1* rs3064744, and *TYMS* rs11280056 was developed, validated, and applied for genotyping PDAC patients’ DNA samples. The genes and polymorphisms were selected as being potentially predictive of 5-fluorouracil, leucovorin, irinotecan, and oxaliplatin toxicity and were analyzed according to probable biological function of the genes as well as the clinical annotations reported in the PharmGKB database (www.pharmgkb.org (accessed on 17 September 2021)).

The genetic variations and genotypes analyzed by the microarray are listed in Table 2.

**Figure 1:**
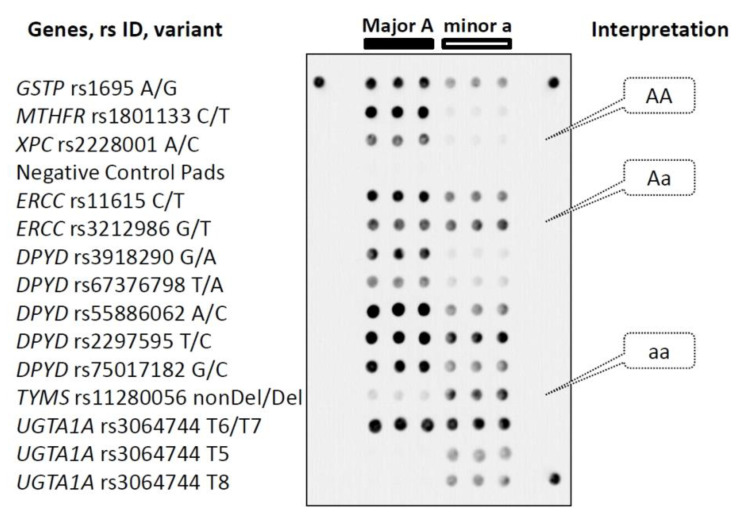
Layout of the genotyping microarray and an example of interpretation of hybridization images. Every horizontal row of gel elements corresponds to the gene variant indicated on the left. Each gel pad harboring a unique interrogating oligonucleotide probe is in triplicate. Gel elements corresponding to the major (A) and minor (a) alleles are arranged in triple left and right columns, respectively. Illustration of identification of homo- and heterozygote genotypes is in the right callouts. Three peripheral gel spots are positioning markers. See the text for details.

The microarray contained three identical gel pads for each polymorphic allele and six gel pads containing no probes to estimate background fluorescence signals. The microarray layout and an example of a hybridization image are shown in Figure 1. The gel pads were arranged in six vertical columns. The first three columns contained probes specific to the major alleles, and the last three included probes to the minor allele variants. Horizontal rows corresponded to the individual polymorphisms.

Genotyping analysis performed on the microarray was based on differences in fluorescence signals acquired from gel pads relevant to the corresponding target polymorphism. The difference occurs because the fluorescently labeled amplification products form perfect hybridization duplexes only with probes that are fully complementary to the target sequences. Otherwise, if the target contains minor polymorphisms (SNP, deletion), a perfect duplex turns into an imperfect duplex, resulting in a significant decrease in the intensity of the corresponding fluorescence signal. The specificity of polymorphism identification by the interrogating oligonucleotide probes was verified by hybridization of pre-sequenced samples. The control DNA samples harboring all allele variations were analyzed by hybridization. Relevant fluorescence signals corresponding to all target polymorphisms were observed for every control sample that confirmed the ability of the developed technique to identify the genetic variants accurately.

### 3.3. Genotyping of Clinical Samples

A total of 78 DNA samples isolated from PDAC patients’ blood that were treated with first-line FOLFIRINOX were genotyped with the microarray-based technique described above. Genotyping was aimed at identification of 12 polymorphisms (SNV and deletions) in 7 genes: *DPYD* (rs3918290, rs75017182, rs55886062, rs67376798, and rs2297595); *XPC* (rs2228001); *GSTP1* (rs1695); *MTHFR* (rs1801133); *ERCC1* (rs3212986 and rs11615); *UGT1A1* (rs3064744); and *TYMS* (rs11280056). A complete set of associations between each polymorphism and adverse events were considered. In particularly, hematological toxicity included anemia, leukopenia, neutropenia, and thrombocytopenia; gastrointestinal toxicity included diarrhea, vomiting, nausea, stomatitis, and mucositis; neurological toxicity included ototoxicity, central neurotoxicity, and peripheral sensory/motor polyneuropathy. Skin and hepatic toxicities were also monitored. Some other patients’ clinical indicators including age, gender, cancer staging, comorbidity, and specific markers (carcinoembryonic (CEA) and carbohydrate (CA19-9) antigens) were taken into consideration.

#### 3.3.1. Neurological Toxicity

Among 78 PDAC patients administered with FOLFIRINOX chemotherapy, neurological toxicity occurred in 25 (32%) patients (Table 3). In all cases, neurotoxicity manifested as peripheral neuropathy.

It was found that the allele 6 bp-deletion of the thymidylate synthase gene (*TYMS* rs11280056) predicted grade 1–2 neurological toxicity in advanced PDAC patients (*p* = 0.0072 and *p* = 0.0019, according to co-dominant and recessive model, respectively).

The homozygous *TYMS* rs11280056 6 bp-deletion genotype was identified in 6/25 (24%) patients and the heterozygous genotype was observed in 10/25 (40%) patients with neurological toxicity. The heterozygous *TYMS* rs11280056 genotype was also identified in 24/53 (45%) patients who did not have any AEs including neurological toxicity.

It was reported that one PDAC patient carrying the homozygous *TYMS* rs11280056 6 bp-deletion genotype had no drug–induced neurological toxicity. During retrospective analysis, we found that the patient had received only three cycles of therapy because of tumor progression.

Thus, patients carrying *TYMS* rs11280056 6 bp-deletion homozygous genotype were at significantly increased risk for grades 1–2 neurological toxicity compared with wild type patients without the deletion.

The genetic variants of the *DPYD*, *XPC*, *GSTP1*, *MTHFR*, and *UGT1A1* genes were not associated with neurotoxicity.

#### 3.3.2. Hematological Toxicity

Differential associations with grade 3–4 hematological toxicity were seen across the treatment arms. Anemia, leukopenia, neutropenia, and thrombocytopenia were pooled into one group, and any of these events was considered as the occurrence of hematological toxicity.

Grade 3–4 hematological toxicity was reported in 31/78 (40%) PDAC patients treated with FOLFIRINOX (Table 3). The presence of the homozygous AA genotype (*GSTP1* rs1695) was observed in 13/31 (42%) patients with hematological adverse events. The heterozygote AG was identified in 16/31 (52%) patients. Only 2/31 (6%) patients with hematological toxicity were homozygous GG genotype carriers.

In a cohort of 47 patients without any hematological toxicity, the allele distribution was as follows: 19/47 (40%) patients harbored the homozygous AA genotype, 15 (32%) patients had the heterozygous AG, and 13 (28%) patients carried the homozygous GG genotype.

According to co-dominant and recessive models, it was found that the GG genotype (also known as GSTP1*B) was associated with a lower risk for grade 3–4 hematological toxicity in advanced PDAC patients (*p* = 0.032 and *p* = 0.014, respectively).

We did not observe any associations between hematological toxicity and other genetic variants in the *DPYD*, *XPC*, *GSTP1*, *MTHFR*, and *UGT1A1* genes.

#### 3.3.3. Allele Frequencies

Allele frequencies obtained by microarray genotyping are summarized in Table 2 and were calculated in a cohort of 78 patients who were representatives of the Eastern Slavs to a great extent. Alleles were designated as major and minor variants according to the 1000 Genomes Project allele’s frequencies for the global population. The allele frequencies obtained from the analyzed samples were comparable to the relevant data of the 1000 Genomes Project (http://www.internationalgenome.org/data (accessed on 7 September 2021)).

## 4. Discussion

Most cytotoxic anticancer drugs are characterized by a dose-related effect and a narrow therapeutic index, that requires AEs monitoring and individual dose selection in some patients. Dose selection is usually based on age and body surface area (BSA) with or without including Therapeutic Drug Monitoring (TDM). However, these characteristics are not always sufficient to determine an optimal therapeutic dose since inter-individual variations remain beyond consideration. For example, in spite of the fact that 5-fluorouracil (5-FU) administration is individualized to the extent of BSA-based dosing, BSA does not correlate accurately with any pharmacokinetic parameters in adults [15]. Thus, pharmacogenomic, pharmacokinetic, and pharmacodynamic factors should be taken into consideration to optimize the benefit for patients, in terms of both antitumor activity and treatment tolerance.

In the current study, we retrospectively evaluated associations between AEs and polymorphic variation in the genes involved in drug metabolism, DNA repair and replication in PDAC patients treated with FOLFIRINOX chemotherapy.

The genetic variants selected as potential predictors of toxicity induced by combination therapy of FOLFIRINOX were as follows: *DPYD* rs3918290, rs75017182, rs55886062, rs67376798, rs2297595, *XPC* rs2228001, *GSTP1* rs1695, *MTHFR* rs1801133, *ERCC1* rs3212986, rs11615, *UGT1A1* rs3064744, and *TYMS* rs11280056. Genotyping was performed on the specialized microarray that was developed and validated in the current study. The approach utilizes a low-density hydrogel microarray platform that had been successfully applied in a number of diagnostic applications (for review, see [16,17]).

The present study is the first report demonstrating the significant association between *TYMS* genetic polymorphism, specifically *TYMS* rs11280056, and neurotoxicity among pancreatic cancer patients treated with FOLFIRINOX. To the best of our knowledge, this association has not been reported earlier. The increased risk for grade 1–2 neurotoxicity was disclosed in PDAC patients with 6 bp-deletion in the *TYMS* gene compared with patients without the deletion (*p* = 0.0072 and *p* = 0.0019, according to co-dominant and recessive model, respectively). Notably, grade 3–4 neurotoxicity has not been reported in the course of the study due to a prompt dose reduction immediately following the early signs of neurological toxicity.

The thymidylate synthase (TYMS) gene is a prospective marker of clinical response and toxicity to 5-FU since this enzyme is a molecular target of fluoropyirimidines [18,19], and polymorphisms in the regulatory regions of the *TYMS* gene may affect its level of expression [20,21]. However, a number of studies contain conflicting results that do not allow proper evaluation the clinical value of these allele variants [22,23,24]. Particularly, some studies report significant association between the clinical response and/or AEs and variations in the *TYMS* 5′UTR and 3′UTR regulatory regions, while other studies contain contradictory results [25,26,27,28].

Notably, Castro-Rojas C. et al. reported that the *TYMS* rs45445694 polymorphism (5’VNTR 2R/2R) was associated with severe toxicity to the 5-FU-based chemotherapy in colorectal cancer patients. In their case, the main types of toxicity were gastrointestinal (46%) and, specifically, neurological toxicity (38%). The latter manifested as dysesthesia and sensory neuropathy [29].

Based on the results of the above study and our findings, it can be assumed that *TYMS* genetic polymorphisms probably could be associated with *fluoropyrimidine*-induced neurotoxicity in other cancer types as well.

Another noteworthy result of the study was that the *GSTP1* rs1695 homozygous GG genotype was associated with a decreased risk for grades 3–4 hematological toxicity compared with the heterozygous AG allele variant and the homozygous AA type (*p* = 0.032 and *p* = 0.014, according to co-dominant and recessive models, respectively).

GSTP1 (Glutathione S-Transferase) encoded by the *GSTP1* gene is an enzyme that plays an important role in the detoxification of xenobiotics, including platinum-based anticancer drugs [30,31]. *GSTP1* is considered to be an indicator of response to chemotherapy and drug-induced AEs [32], although no definite conclusions have been derived.

A number of studies have reported the association between *GSTP1* rs1695 and hematological toxicity, and patients carrying the homozygous GG genotype (GSTP1*B) had a decreased risk for drug-induced hematological toxicity [33,34]. The results obtained in the current study fit fairly well with these findings. We have found that among 31 patients with hematological toxicity only 2 (6%) carried the GG genotype. By contrast, in the patient cohort without hematological toxicity, 13/47 (28%) patients harbored the GG genotype. Nevertheless, we suppose the rs1695 GG genotype probably should not be considered a strong predictive biomarker for a lower risk for hematological toxicity, due to relatively high *p*-values and the limited sample size. The impact of this allele variant requires further investigation in a larger sample size by well-designed prospective clinical trials.

Besides the gene variations mentioned above, we have analyzed other genetic variants in the *DPYD*, *UGT1A1*, *ERCC1*, *XPC*, and *MTHFR* genes. We did not find any associations between AEs and genetic polymorphisms in these genes.

*DPYD* and *UGT1A1* were included in the genotyping microarray since these genes encode two key drug metabolizing enzymes, dihydropyrimidine dehydrogenase (DPD) and uridine diphosphate glucuronosyltransferases (UGT1A1), that are involved in the catabolic pathways of 5-FU and irinotecan [35,36]. Genotyping of germline variations in these genes is strongly required to establish an effective dose of 5-FU and irinotecan, respectively.

According to the Guideline for Fluoropyrimidines and DPYD issued by Clinical Pharmacogenetics Implementation Consortium (CPIC), the frequency of *DPYD* allele variants significantly associated with decreased function of the enzyme is low enough and in most variants does not exceed <0.005 [37]. Nevertheless, deficiency in the DPD enzyme leads to increased exposure to the cytotoxic agent and its active metabolites and, consequently, an increased risk of related AEs.

The most well-studied genetic variants of *UGT1A1* are UGT1A1*28 and UGT1A1*6. The UGT1A1*28 gene variant is a tandem TA repeat polymorphism [A(TA)7TAA] in the *UGT1A1* promoter region that leads to decreased gene expression. Homozygous carriers of the variants T7/T7 (patients with so called Gilbert’s syndrome) have decreased UGT1A1 expression by approximately 70% [38,39].

The *ERCC1* and *XPC* gene polymorphisms were analyzed since the genes are involved in the nucleotide-excision repair (NER) DNA-repair pathway. Excision repair cross-complementation group 1 (ERCC1) encoded by the *ERCC1* gene is a key protein in the NER pathway. Together with xeroderma pigmentosum complementation group F (XPF), ERCC1 forms a heterodimer complex and participates in the elimination of different DNA adducts induced by UV light, reactive oxygen species, environmental mutagens, and especially cancer chemotherapy drugs during NER [40,41]. The XPF/ERCC1 complex is also involved in double strand break repair (DSBR) [42].

Multiple studies are devoted to investigating polymorphisms in the *ERCC1 gene* (e.g., see review [43]). The most commonly investigated SNV in the *ERCC1* gene is rs11615. The majority of studies reported a significant association between the mutant CC genotype and better DFS, PFS, and OS [44,45,46,47,48,49]. However, some studies showed contradictory results and reported that patients with the CC genotype had worse treatment outcome in terms of PFS and OS [50,51,52].

Another primary NER initiating protein is the xeroderma pigmentosum group C (XPC). This protein plays an essential role in the first steps of NER, particularly in damage recognition as well as open complex formation and reparation [53,54,55]. It was reported that SNV in the *XPC* gene are potential markers of treatment response to oxaliplatin-based therapy in cancer patients [56,57,58]. Therefore, genetic variations in *ERCC1* and *XPC* may have prospective value for predicting response to oxaliplatin-based chemotherapy, and further investigation of these polymorphisms seems essential and reasonable.

Methylenetetrahydrofolate reductase (MTHFR) encoded by the *MTHFR* gene is the most critical enzyme in the metabolism of folate and fluoropyrimidines [59]. Therefore, the MTHFR enzymatic activity may hypothetically predict the clinical responses and toxicity to 5-FU. One of the most frequent functional SNV in the *MTHFR* gene is rs1801133 (C > T; Ala222Val). This variant occurs in the homozygous state in 10–15% of many North American and European populations and correlates with reduced enzyme activity [59,60]. However, the evidence of genetic association remains relatively weak, and the results reported in systematic reviews are not consistent [61,62,63]. Therefore, further well-designed prospective investigations are needed to validate the present findings.

Multiple studies have reported significant world-wide variation in cancer prognosis between ethnic groups [64,65,66]. In the current study, microarray genotyping resulted in allele frequency determination in a cohort of 78 patients who were representatives of the Eastern Slavs to a great extent. We suppose this data will also contribute to further investigation of drug-induced AEs associated with different ethnic groups.

This study has some strengths and limitations. Our study’s main strength is the identification of the novel association between *TYMS* rs11280056 genetic polymorphism and neurotoxicity that could be taken into consideration for estimation of the increased risk of drug-induced AEs. The microarray developed in the study seems a promising genotyping tool to simultaneously identify a dozen of gene polymorphic variants accurately and relatively quickly (in one day). Additional data related to the impact of the *GSTP1* rs1695 genetic polymorphism on the risk for hematological drug-induced toxicity was also reported.

The present study has some limitations: (i) in statistical analysis, we pooled drug-induced anemia, leukopenia, neutropenia, and thrombocytopenia into one group (hematological toxicity) and did not consider each syndrome separately; (ii) the number of patients enrolled in the study was not very large, and further prospective investigations are desirable to confirm and quantitate the findings; and (iii) every association identified in the current study is related to the whole combination therapy of FOLFIRINOX that includes four chemotherapy agents. We did not investigate the effects of each agent separately.

## 5. Conclusions

In summary, the results of the present study indicate that the *TYMS* genetic polymorphism rs11280056 is significantly associated with grade 1–2 neurotoxicity among pancreatic cancer patients treated with FOLFIRINOX. This association is reported for the first time and *TYMS* rs11280056 polymorphic variant could be considered a promising prognostic marker of neurotoxicity. Another association between the *GSTP1* rs1695 GG genotype and the decreased risk for grade 3–4 hematological toxicity was also identified but it was not as strong and requires further validation.

Further validation studies on a larger number of patients are desirable to confirm and quantitate these associations. It may help clinicians to identify patients who have a high risk of FOLFIRINOX-induced AEs and improve treatment decision for cancer patients.

## Figures and Tables

**Table 1:** Clinical information of PDAC patients.

Patient Characteristics	
Sex, *n* (%)	
Male	37 (47.4%)
Female	41 (52.6%)
Median age, years (Range)	59 (28–77)
T stage, *n* (%)	
T1–T2	7 (9.0%)
T3	31 (39.7%)
T4	40 (51.3%)
Primary tumor diameter, mm	
Median (Range)	40 (0–115)
Primary tumor site, *n* (%)	
Pancreatic head	41 (52.6%)
Pancreatic body-tail	37 (47.4%)
CA 19-9, (IU/mL)	
Median (Range)	317 (1–51,878)
Performance status (ECOG)	
0–1	77 (98.7%)
2	1 (1.3%)
Extent of disease, *n* (%)	
Resectable or borderline resectable	9 (11.5%)
Advanced and metastatic	69 (88.5%)
Indication for chemotherapy, *n* (%)	
Induction with or w/o adjuvant	40 (51.3%)
Adjuvant	1 (1.3%)
First-line in metastatic setting	37 (47.4%)
Number of therapy cycles	
Median (Range)	6 (1–12)
Cancellation reason, *n* (%)	
Progressive disease	14 (17.9%)
Toxicity	6 (7.7%)
Completion of the scheduled number of cycles	51 (65.4%)
Patient refusal	3 (14.1%)
Other	4 (5.1 %)

**Table 2:** Genes, genetic variations, genotype, and allele frequencies in PDAC patients (*n* = 78).

Gene, rs ID Number	Type of Variation	Nucleotide (Amino Acid) Change	Consequence	Genotype Counts (Frequency) *			Allele Frequency *	
				AA	Aa	aa	A	a
*DPYD*, rs2297595	SNV	T > C (Met166Val)	Initiator Codon Variant	56 (0.72)	22 (0.28)	0 (0)	0.86	0.14
*DPYD*, rs3918290	SNV	G > A	Splice Donor Variant	76 (0.97)	2 (0.03)	0 (0)	0.99	0.01
*DPYD*, rs55886062	SNV	A > C (Ile560Ser)	Missense Variant	78 (1)	0 (0)	0 (0)	1.00	0
*DPYD*, rs67376798	SNV	T > A (Asp949Val)	Missense Variant	78 (1)	0 (0)	0 (0)	1.00	0
*DPYD*, rs75017182	SNV	G > C	Intron Variant	77 (0.99)	1 (0.01)	0 (0)	0.99	0.01
*ERCC1*, rs3212986	SNV	G > T (Gln506Lys)	3 Prime UTR Variant	39 (0.5)	34 (0.44)	5 (0.06)	0.72	0.28
*ERCC1*, rs11615	SNV	T > C (Asn118Asn)	Synonymous Variant	27 (0.35)	39 (0.5)	12 (0.15)	0.58	0.42
*GSTP1*, rs1695	SNV	A > G (Ile105Val)	Missense Variant	32 (0.41)	31 (0.4)	15 (0.19)	0.61	0.39
*MTHFR*, rs1801133	SNV	C > T (Ala222Val)	Missense Variant	42 (0.54)	27 (0.35)	9 (0.12)	0.71	0.29
*TYMS*, rs11280056	6 bp deletion	Insertion/ Deletion	3 Prime UTR Variant	37 (0.47)	34 (0.44)	7 (0.09)	0.69	0.31
*UGT1A1*, rs3064744	2 bp insertion	Insertion/ Deletion	Insertion/Del Variation	33 (0.42)	35 (0.45)	10 (0.13)	0.65	0.35
*XPC*, rs2228001	SNV	A > C (Lys939Gln)	Missense Variant	27 (0.35)	40 (0.51)	11 (0.14)	0.60	0.40

*, Frequencies were determined in this study; A: major allele frequency; a: minor allele frequency; SNV: single nucleotide variation; bp: base pair.

**Table 3:** *TYMS* rs11280056 and *GSTP1* rs1695 genotype frequencies identified in PDAC patients treated with FOLFIRINOX (*n* = 78).

Toxicity	Genotype Counts (Frequency)			*p*-Value
***TYMS* rs11280056**				
**Neurological toxicity**				
	nonDel/ nonDel	nonDel/Del	Del/Del	
Grade 1–2 Peripheral neuropathy (*n* = 25)	9 (36%)	10 (40%)	6 (24%)	0.0072 (co-dominant model) 0.0019 (recessive model)
No AEs ^a^ (*n* = 53)	28 (53%)	24 (45%)	1 (2%)	
***GSTP1* rs1695**				
**Hematological toxicity**				
	AA	AG	GG	
Grade 3–4 leukopenia, neutropenia or thrombocytopenia ^b^ (*n* = 31)	13 (42%)	16 (52%)	2 (6%)	0.032 (co-dominant model), 0.014 (recessive model)
No AEs (*n* = 47)	19 (40%)	15 (32%)	13 (28%)	

^a^, Adverse events. ^b^, Leukopenia, neutropenia, and thrombocytopenia were pooled together and reported as hematological toxicity.

## Data Availability

The data that support the findings of this study are available from the corresponding author upon reasonable request.

## Supplementary Materials

The following are available online at https://www.mdpi.com/article/10.3390/pharmaceutics14010077/s1: Table S1: Multiplex PCR primers used to prepare DNA samples for microarray genotyping; Table S2: Sequences of oligonucleotide probes immobilized on the microarray.
