# Supplementary material for: TYMS 3′-UTR Polymorphism: A Novel Association with FOLFIRINOX-Induced Neurotoxicity in Pancreatic Cancer Patients
Source: Pharmaceutics. 2021 Dec 29;14(1):77. doi: 10.3390/pharmaceutics14010077 (PMC8779442; doi:10.3390/pharmaceutics14010077)
Supplement: Supplementary file 1 [file pharmaceutics-14-00077-s001.zip › pharmaceutics-1480840-supplementary.pdf]

pharmaceutics

Supplementary Materials: *TYMS* 3′-UTR Polymorphism: A Novel Association with FOLFIRINOX-Induced Neurotoxicity in Pancreatic Cancer Patients

Marina Emelyanova, Ilya Pokataev, Igor Shashkov, Elena Kopantseva, Vladimir Lyadov, Rustam Heydarov and Vladimir Mikhailovich

Table S1. Multiplex PCR primers used to prepare DNA samples for microarray genotyping.

Primer	Sequence 5` → 3`
DPYD rs2297595_for*	CTCTGCGAGCATAATGTACTTGCAGGCCCAGCACCAAAAAGAGCAAT
DPYD rs2297595_rev	GGCTGTACGCTGTCAGGAGGCATGACTCTAGAAAGGAAAGACTGAAAGTTAGCCA
DPYD rs3918290_for	CTCTGCGAGCATAATGTGGCCCTGGACAAAGCTCCTTTCTGAATA
DPYD rs3918290_rev	GGCTGTACGCTGTCTTCTATGCATCAGCAAAGCAACTGGCAGA
DPYD rs55886062_for	CTCTGCGAGCATAATGGTTTTGGTGAGGGCAAAACCCCATCC
DPYD rs55886062_rev	GGCTGTACGCTGTCGAGCTTCCGTTTCTGCCAAGCCTG
DPYD rs67376798_for	CTCTGCGAGCATAATGAATTCCAGCAGGATTCTTACCTGGTAGCCAGAATCAT
DPYD rs67376798_rev	GGCTGTACGCTGTCAGCACTGCAGTACCTTGGAACATTTGGTGAATTGAGC
DPYD rs75017182_for	CTCTGCGAGCATAATGGGAGCATGTCTTTATGAGGGACAAC
DPYD rs75017182_rev	GGCTGTACGCTGTCTTTCACTCAGCATCAGCCACATATC
ERCC1 rs11615_for	CTCTGCGAGCATAATGAGGGCCCTGTGGTTATCAAGGGTCATCCCTATT
ERCC1 rs11615_rev	GGCTGTACGCTGTCTGCTCTGGCCCAGCACATAGTCGGGAA
ERCC1 rs3212986_for	CTCTGCGACCATAATGGGCACCTTCAGCTTTCTTTAGTTCCTCAGT
ERCC1 rs3212986_rev	GGCTGTACGCTGTCAGGAGATGCCAGGGCCGCCACT
GSTP1 rs1695_for	CTCTGCGAGCATAATGAGGGCTCTATGGGAAGGACCAGCAGGA
GSTP1 rs1695_rev	GGCTGTACGCTGTCGTGCCCAACCCTGGTGCAGATGCTCA
MTHFR_rs1801133_for	CTCTGCGAGCATAATGCGAAGCAGGGAGCTTTGAGGCTGACCTGAAG
MTHFR_rs1801133_rev	GGCTGTACGCTGTCTCGGTGCATGCCTTCACAAAGCGGAAGAA
TYMS rs11280056_for	CTCTGCGAGCATAATGCTGAGGGAGCTGAGTAACACCATCG
TYMS rs11280056_rev	GGCTGTACGCTGTCGGAAGGAACTGAGCAGATAAGTGGC
UGT1A1 rs3064744_for	CTCTGCGAGCATAATGCTCTGAAAGTGAACTCCCTGCTACCTTTGT
UGT1A1 rs3064744_rev	GGCTGTACGCTGTCCTTTGCTCCTGCCAGAGGTTC
XPC_rs2228001_for	CTCTGCGAGCATAATGAAAGCAGAAGCTGAAGGGTGGGCCCAAGAA
XPC_rs2228001_rev	GGCTGTACGCTGTCCGCCTCCGTGCATGCTGCCTCAGTTT
Adapter_for	CTCTGCGAGCATAATG
Adapter_rev	GGCTGTACGCTGTC

* for and rev, forward and reverse primers, respectively.

Table S2. Sequences of oligonucleotide probes immobilized on the microarray.

Oligonucleotide Probe	Sequence 5` → 3`
DPYD rs2297595 C	TGTGGGATACTCACTGCTTTGAATAC*
DPYD rs2297595 T	CTGTGGGATACTCATTGCTTTGAATAC
DPYD rs3918290 A	TTCCAGACAACATAAGTGTGAT
DPYD rs3918290 G	CCAGACAACGTAAGTGTGA
DPYD rs55886062 C	AGCTCTTCGACTCATTGATGTG
DPYD rs55886062 A	AGCTCTTCGAATCATTGATGTGC
DPYD rs67376798 A	CATTTCTTCAACAATCATAGCC
DPYD rs67376798 T	CATTTCTTCATCAATCATAGCC
DPYD rs75017182 G	TTCTCAGCTTTGATTTTCAC
DPYD rs75017182 C	AATTCTCACCTTTGATTTTCAC
ERCC1 rs11615 C	GTGCGCAACGTGCCCT
ERCC1 rs11615 T	GTGCGCAATGTGCCCTG
ERCC1 rs3212986 G	CTGCTGCTGCTTCCGCTTCTT
ERCC1 rs3212986 T	GCTGCTGCTTCTTCCGCTTCTT
GSTP1 rs1695 A	CTGCAAATACATCTCCCTCAT
GSTP1 rs1695 G	TGCAAATACGTCTCCCTCA
MTHFR rs1801133 T	TGCGGGAGTCGATTTCA
MTHFR rs1801133 C	TGCGGGAGCCGATTTCAT
TYMS rs11280056 Del	GTGGTTATGAACTTTATAGTTGTTT
TYMS rs11280056 Non-Del	GTGGTTATGAACTTTAAAGTTATAG
UGT1A1 rs3064744 T5	GCCATATATATATATATAAG
UGT1A1 rs3064744T6	GCCATATATATATATATATAAG
UGT1A1 rs3064744 T7	GCCATATATATATATATATATAAG
UGT1A1 rs3064744 T8	GCCATATATATATATATATATATAAG
XPC rs2228001 A	TTCCCATTTGAGAAGCTGTGAG
XPC rs2228001 C	TTCCCATTTGAGCAGCTGTGA

* All hybridization probes contained 3′-Amino-Modifier C6 CPG 500 for copolymerization with microarray hydrogel components. For details, see the text.
